# Elongation rate of RNA polymerase II affects pausing patterns across 3′ UTRs

**DOI:** 10.1016/j.jbc.2023.105289

**Published:** 2023-09-24

**Authors:** Alexandra Khitun, Christian Brion, Zarmik Moqtaderi, Joseph V. Geisberg, L. Stirling Churchman, Kevin Struhl

**Affiliations:** 1 Departments of Biological Chemistry and Molecular Pharmacology, Harvard Medical School, Boston, Massachusetts, USA; 2 Department of Genetics, Harvard Medical School, Boston, Massachusetts, USA

**Keywords:** transcription, RNA polymerase II, RNA synthesis, polyadenylation, gene regulation

## Abstract

Yeast mRNAs are polyadenylated at multiple sites in their 3′ untranslated regions (3′ UTRs), and poly(A) site usage is regulated by the rate of transcriptional elongation by RNA polymerase II (Pol II). Slow Pol II derivatives favor upstream poly(A) sites, and fast Pol II derivatives favor downstream poly(A) sites. Transcriptional elongation and polyadenylation are linked at the nucleotide level, presumably reflecting Pol II dwell time at each residue that influences the level of polyadenylation. Here, we investigate the effect of Pol II elongation rate on pausing patterns and the relationship between Pol II pause sites and poly(A) sites within 3′ UTRs. Mutations that affect Pol II elongation rate alter sequence preferences at pause sites within 3′ UTRs, and pausing preferences differ between 3′ UTRs and coding regions. In addition, sequences immediately flanking the pause sites show preferences that are largely independent of Pol II speed. In wild-type cells, poly(A) sites are preferentially located < 50 nucleotides upstream from Pol II pause sites, but this spatial relationship is diminished in cells harboring Pol II speed mutants. Based on a random forest classifier, Pol II pause sites are modestly predicted by the distance to poly(A) sites but are better predicted by the chromatin landscape in Pol II speed derivatives. Transcriptional regulatory proteins can influence the relationship between Pol II pausing and polyadenylation but in a manner distinct from Pol II elongation rate derivatives. These results indicate a complex relationship between Pol II pausing and polyadenylation.

RNA polymerase II (Pol II) transcriptional initiation and elongation regulate co-transcriptional processes such as splicing and polyadenylation that create transcriptome diversity (1, 2, 3, 4, 5). Pol II elongation speed is regulated within genes, across different genes, and in response to environmental stimuli (2, 6, 7). Elongation speed affects the exposure rate of mRNA sequences that could serve as protein binding sites and recruit 3′-end processing factors that are in kinetic competition with elongating Pol II (4, 8). Elongation rate affects Pol II dwell time at individual nucleotides, thereby affecting the rate of co-transcriptional processes throughout the 3′UTR. Pol II speed therefore influences the relative levels of 3′ isoforms (3, 5, 9, 10, 11), although the mechanisms that underlie this regulation are not well defined.

Essentially all eukaryotic genes produce numerous 3′ mRNA isoforms through alternative polyadenylation at multiple sites located within the 3′UTRs (12, 13, 14). Although 3′ isoforms typically, but not always, encode the same protein, structural and sequence elements in the 3′UTR can control biological functions including transcript stability, translation, and transport (15, 16, 17). Slow Pol II elongation is linked to preference for proximal poly(A) sites in yeast and flies (3, 5, 9, 10, 11). Conversely, fast Pol II elongation in yeast shifts polyadenylation towards distal sites (3, 5). Notably, these Pol II speed mutants shift the relative abundance of isoforms, but they do not generate new poly(A) sites.

The association between shifts in poly(A) sites and Pol II elongation rate is primarily based on assays that measure steady-state mRNA levels. As Pol II elongation is a dynamic process, techniques that directly profile Pol II behavior at various elongation speeds are important for understanding how elongation rate modulates poly(A) site selection. Native elongating transcript sequencing (NET-seq) is a genome-wide, nucleotide-resolution technology that tracks Pol II occupancy across transcribed regions (18). NET-seq reveals Pol II pause sites as high signals at specific nucleotide locations as compared with average signals throughout the gene. Many factors contribute to Pol II pausing, including DNA sequence and shape, chromatin landscape, phosphorylation state of the Pol II C-terminal domain (CTD), and elongation factors (19). As such, the transcriptional elongation rate may influence Pol II pausing in some contexts and not others, therefore affecting downstream RNA processing including polyadenylation and splicing. Previous NET-seq analyses did not analyze Pol II speed mutants, focused on the gene body, and only mapped pauses with respect to the most highly utilized site of polyadenylation (19).

Here, we investigate the relationship between Pol II pausing and poly(A) site selection using yeast strains bearing single amino acid mutations in Rpb1, the largest Pol II subunit, that cause altered Pol II elongation rate. We show that Pol II elongation speed influences pause sequence preferences, accumulation of Pol II signal near annotated poly(A) sites, and the close co-occurrence of 3′ UTR pauses and poly(A) sites.

## Results

### Elongation rate alters Pol II pausing in the 3′ UTR

**Figure 1:**
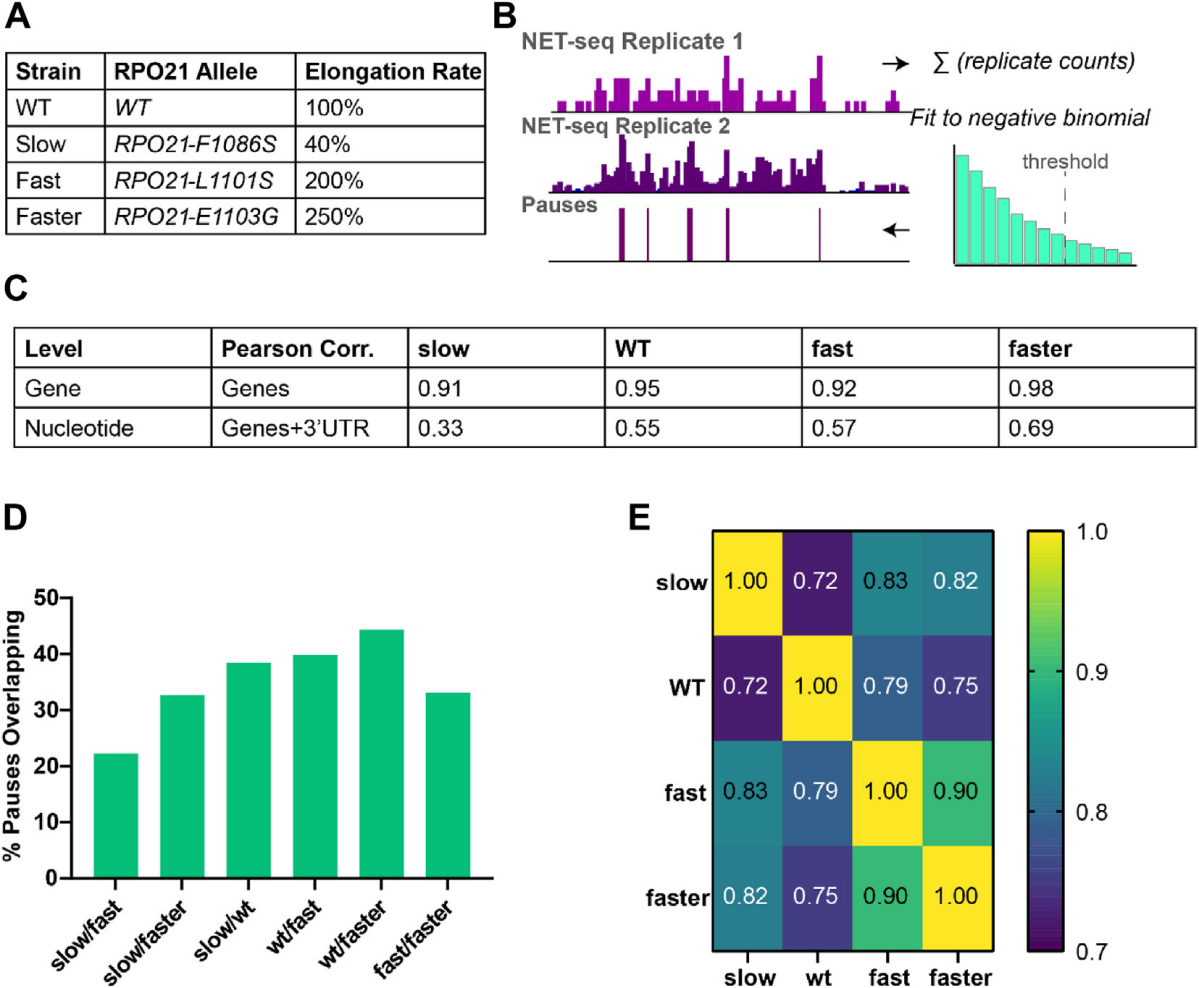
**Pausing in Pol II mutants with slow and fast elongation speeds**. *A*, Pol II derivatives and resulting elongation rate phenotype. *B*, schematic for calling Pol II pauses. NET-seq reads from two well-correlated biological replicates are summed and filtered using a coverage threshold at the gene level. Pauses are identified as nucleotides with read densities higher than three standard deviations above the mean of the adjacent 200 nt as determined by fitting read counts to a negative binomial distribution. *C*, NET-seq data reproducibility. Pearson correlation coefficients between biological replicates of NET-seq data calculated at either the gene level using RPKM values (*top row*) or at nucleotide level using raw read counts within high coverage (>2 reads/nt) genes (*bottom row*). 3′ UTRs are defined as the 400 nt downstream of the stop codon. *D*, percentage of maximal overlap between nucleotide-resolution pauses identified in each Pol II mutant strain. *E*, pearson correlation matrix of weighted average 3′ UTR pause positions across all genes passing the minimum coverage threshold.

Yeast strains that bear point mutations in the trigger loop region of Rpb1 can exhibit slower or faster Pol II elongation rates than wild-type Pol II (20) (Fig. 1*A*). In previous work, we analyzed yeast strains expressing Slow, Fast, and Faster Pol II derivatives for their effect on poly(A) site selection (3, 5). Here, we investigate the effect of elongation rate on Pol II pausing profiles in the 3′UTR by generating derivatives of these strains that contain a 3x-FLAG epitope tag C-terminal to Rpb3. NET-seq analyses of the resulting strains (an example locus in Fig. 1*B*) show excellent reproducibility of biological duplicates at the gene level (Pearson *R* ≥ .91), where counts are summed across an entire gene and correlated between replicates. A more modest but significant correlation is observed at the nucleotide level (Pearson *R* = .33–.69), where counts at each nucleotide position are correlated between biological replicates within defined genomic regions that include a 400 nt 3′ UTR window (Fig. 1*C*). This relatively modest nucleotide-level correlation is likely due to counting error at genomic regions with low sequencing coverage (21).

As sequence coverage at the nucleotide level is low, we combined raw read counts from biological replicates before subjecting the data to thresholding based on coverage and pause calling. Pauses are called at positions with read counts that exceed three standard deviations above the surrounding 200 nucleotides, and are restricted to genes with a minimal coverage of two reads per nucleotide. Although this method precludes direct comparison of pause locations between replicates, the percentage of overlapping pause positions between strains (22–44% overlap, Fig. 1*D*) is comparable to previously reported data reproducibility for biological replicates in the same strain (19). Pause distributions evaluated as a weighted average across 3′UTRs are well correlated among all strains (Fig. 1*E*). Importantly, the correlations of the wild-type strain with any of the mutant strains (Pearson R ranging between 0.72 and 0.79) are lower than the correlations between any two mutant strains, suggesting that elongation rate alters Pol II pausing within the 3′UTR. Fast and Faster Pol II derivatives show the highest level of similarity between weighted average pause positions in the 3′ UTR (Pearson *R* = .90), consistent with their similar elongation phenotypes.

### Pol II speed mutants have different pausing sequence preferences in the 3′UTR

**Figure 2:**
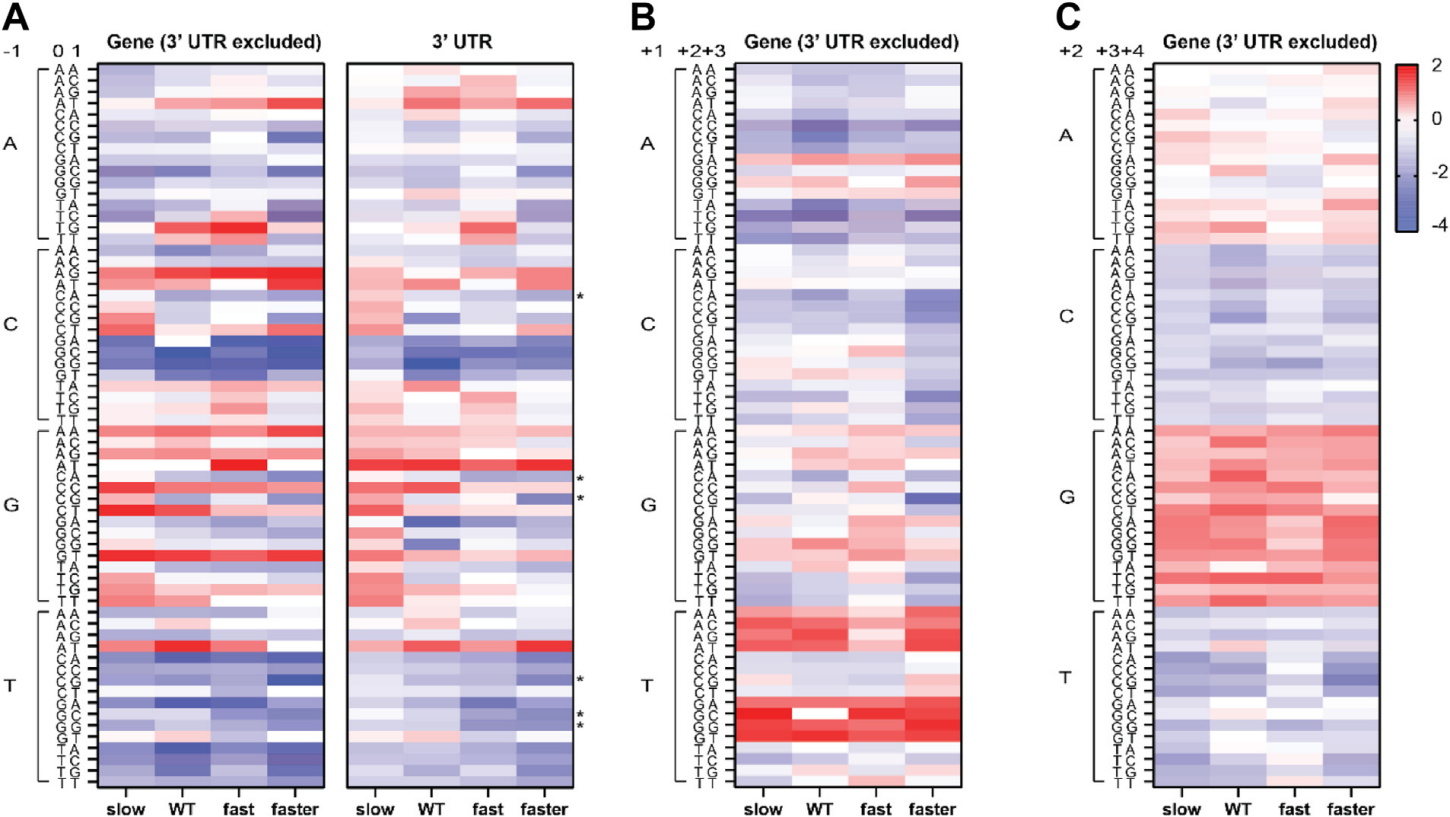
**Pol II elongation speed affects pausing sequence preferences within genes and in 3′ UTRs**. Position 0 is the pause site and positions −1 and +1 are the upstream and downstream flanking nucleotides, respectively. Heatmap colors represent the log_2_ of the ratio between real and shuffled pause frequency at every possible trinucleotide at the following positions: (*A*) −1 to +1; (*B*) +1 to +3; (*C*) +2 to +4). Trinucleotides, where pause enrichment correlates with Pol II speed, are highlighted with an *asterisk* in (*A*).

We used trinucleotide sequence analysis to define sequences that favor Pol II pausing. To avoid biases related to different numbers of identified pauses (pause density), a set of shuffled control pauses were generated for each condition and used to normalize the data. Figure 2*A* shows that trinucleotide sequences surrounding Pol II pause sites (positions −1, 0, and +1) can be either favored (red) or disfavored (blue). Such strong sequence preferences are not observed when comparing two independent sets of shuffled control pauses (Fig. S1). Interestingly, Pol II speed mutants show distinct trinucleotide sequence preferences at pause sites that are different from wild-type and from one another (Fig. 2*A*). In the Slow Pol II strain, there is a modest preference to pause one nucleotide downstream of a G nucleotide. In wild-type, Fast, and Faster strains, a G at the −1 nucleotide is less favored. Additionally, 3′ UTR pauses with T at the −1 position are less favored with increasing elongation speed at some trinucleotides (TCG, TGA, TGC, and TGG). At other trinucleotides (*e.g.*, CCA), pausing is relatively enriched in the 3′ UTR in the Slow Pol II strain and disfavored in the Fast and Faster strains.

As Pol II contacts DNA beyond the active site, we extended this trinucleotide analysis to regions at positions −4 to +4 with respect to pause sites. There is a striking preference for a TG and TA at the +1 and +2 positions (Figs. 2, *B* and *C* and S2) in all four strains. In addition, there is a preference for some trinucleotides with an A at the −4, −3, and −2 positions in all strains (Fig. S2). These strain-independent preferences at more distal positions from the pause site are in contrast from those at the −1, 0, and +1 positions, which can differ among the strains.

### Pausing differences between the gene body and 3′UTR

**Figure 3:**
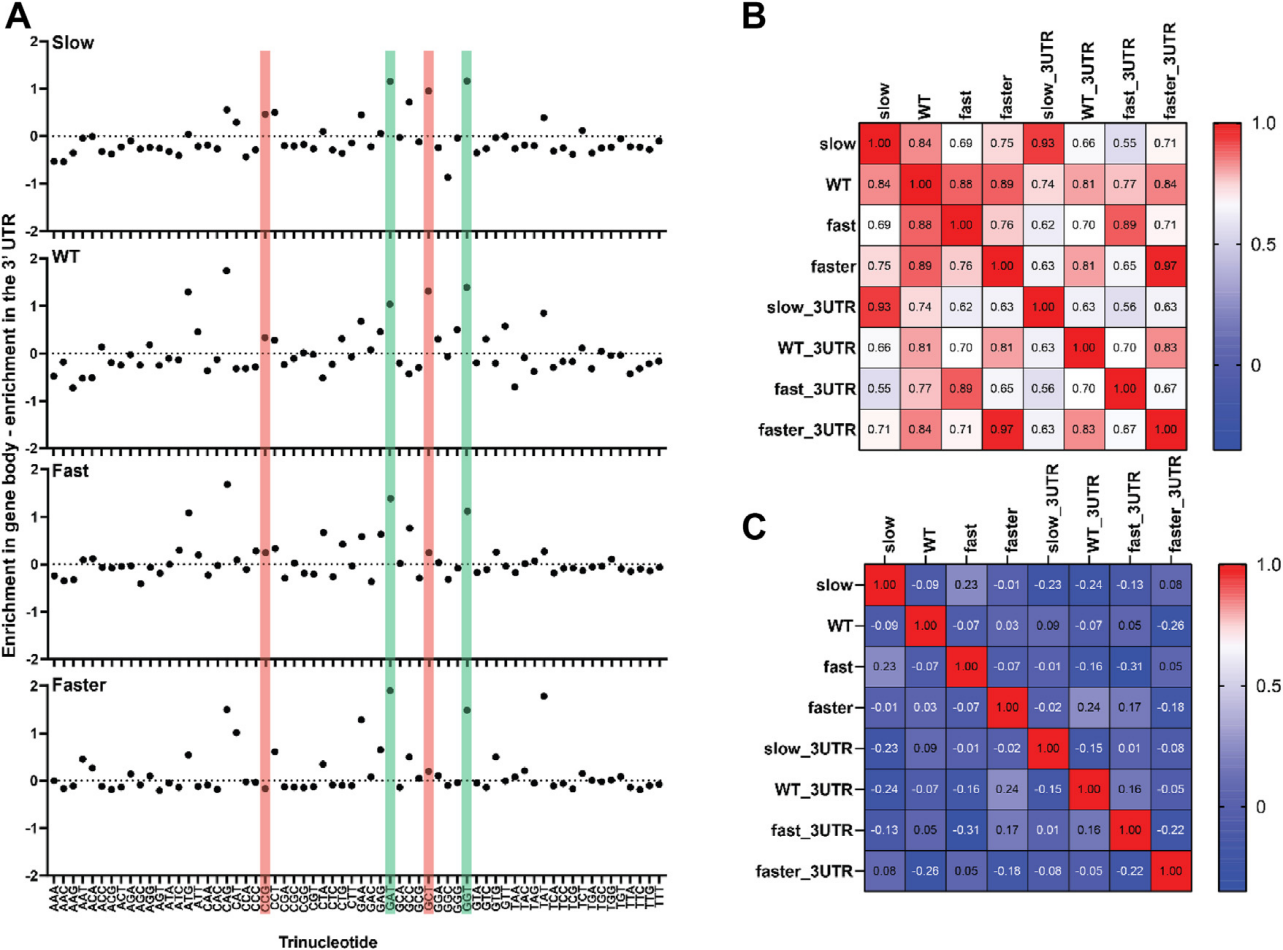
**Pausing sequence preferences track with Pol II speed.***A*, the effect of primary sequence on pausing frequency in the gene body vs. the 3′ UTR. The ratio of real to shuffled pauses in the 3′ UTR was subtracted from the ratio of real to shuffled pauses in the gene body for every trinucleotide sequence. A positive value indicates a trinucleotide is a more favorable pause sequence in the gene body compared to the 3′ UTR and vice versa. *Red bars* highlight trinucleotides where Pol II pausing changes with Pol II speed. *Green* bars highlight trinucleotides where Pol II pausing is not sensitive to speed-associated mutations. *B*, Pearson correlation matrix of trinucleotide enrichments in pauses across all strains. The number of real pauses at each trinucleotide was divided by the number of shuffled control pauses at that trinucleotide. These normalized ratios were compared across strains and summarized using a Pearson correlation coefficient. Pauses localized to the 5′ UTR and within the coding sequence (top four rows) or the 3' UTR (bottom four rows) of well-expressed genes were included. The number of pauses within the gene body (and 3′ UTR) for the different strains are as follows: Slow, 25,308 (10,365); Wild-type, 5755 (2823); Fast, 23,227 (8092); Faster, 61,970 (18,348). *C*, the correlation in part (*B*) was repeated for two sets of shuffled pauses.

We compared sequence preferences at pause sites (positions −1, 0, and +1) between the gene body and the 3′UTR. For most trinucleotides, Pol II pausing preferences are similar between the gene body and the 3′UTR (Fig. 3*A*). However, pausing at GAT and GGT is more frequent in the gene body across all four strains (green bars). In contrast, pauses at CCG and GCT are less frequent in the gene body as Pol II speed increases (red bars). Trinucleotide preferences are more distinct between strains (Pearson R ranging from 0.56 to 0.83 across 3′ UTR pauses) than in different gene regions of a single strain (Pearson R ranging between 081 and 0.97) (Fig. 3*B*). These strong correlations are not observed when comparing two sets of randomly shuffled control pauses localized to the same genomic regions (Fig. 3*C*). Trinucleotide sequence preferences at positions flanking Pol II pause sites are similar at gene bodies and 3′ UTRs (Fig. S2).

### Polyadenylation sites are non-randomly distributed with respect to Pol II pauses

**Figure 4:**
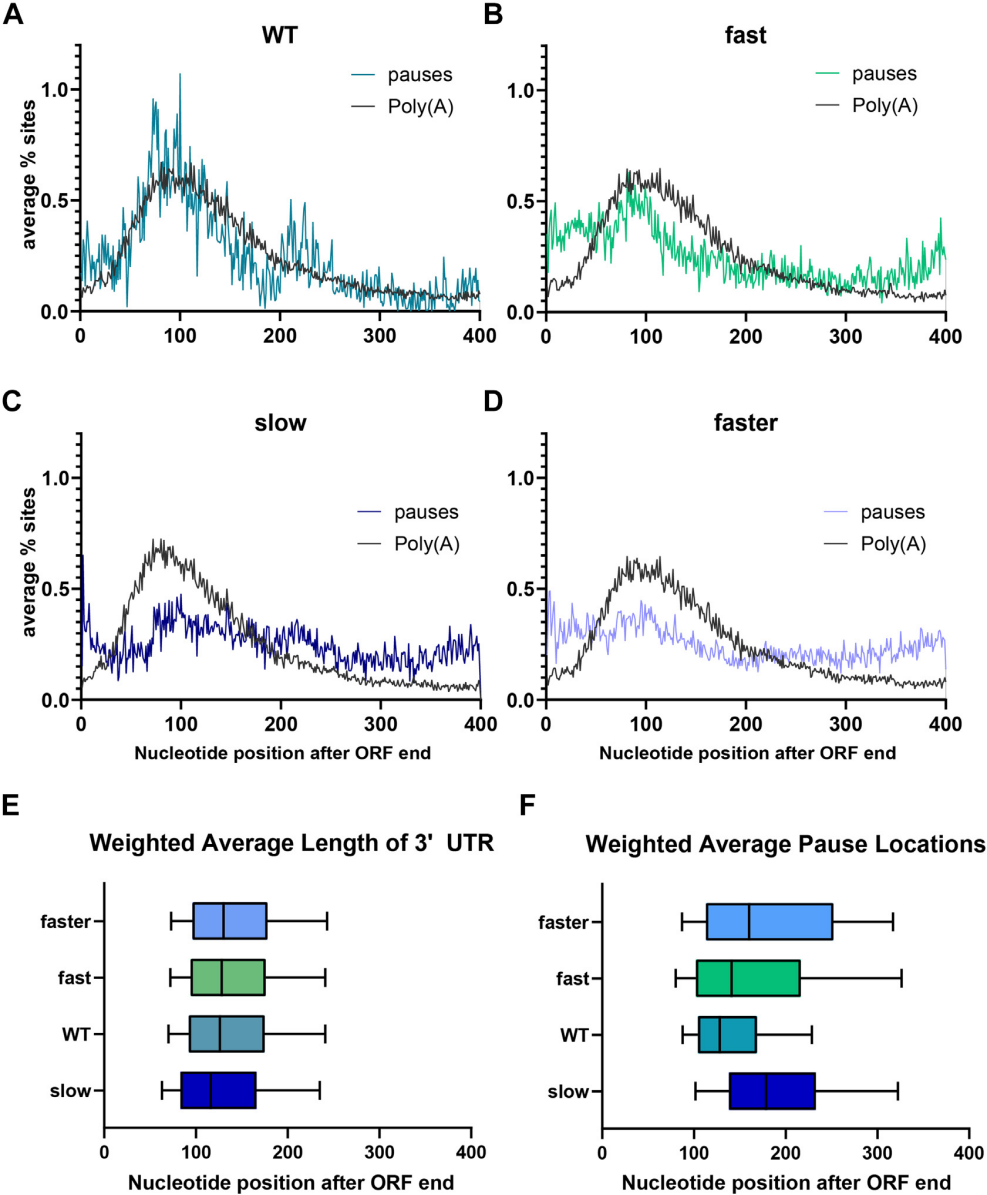
**Distribution of Pol II pauses and polyadenylation signals within 3′ UTRs in each Pol II mutant**. *A–D*, colored line graphs depict the average percentage of pause-associated nucleotides at each position 1 to 400 nt downstream of the stop codon, while the *black* line depicts the average percentage of poly(A) site-associated nucleotides at each position. Data represent combined biological replicates of NET-seq or 3′ READs data, respectively. *E* and *F*, weighted average locations of poly(A) sites (*E*) and pause sites (*F*) relative to the stop codon across yeast genes; poly(A) site data from (3, 5). Whiskers represent 10th to 90th percentile of the data with the median value indicated as a vertical line.

To investigate Pol II pausing positions relative to the profile of poly(A) sites in the same strains, we plotted the average percentage of Pol II pauses and polyadenylation sites across 3′ UTRs (Fig. 4, *A*–*D*). We used quantitative, nucleotide-resolution poly(A) profiles at all positions 400 nt downstream from the stop codon of each gene as well as nucleotide-resolution pause data. Wild-type Pol II shows a prominent pause signature near nucleotides with highest rates of polyadenylation (∼100 nt downstream of the stop codon; Fig. 4*A*). This relationship between Pol II pausing and poly(A) sites is impaired in Rpb1 derivatives with altered elongation rates, in which pauses are more evenly distributed throughout the 3′ region (Fig. 4, *B*–*D*).

Pol II speed mutants change the relative utilization, but not the location, of poly(A) sites (3). Specifically, slow elongation rate causes an upstream shift in poly(A) sites, whereas fast elongation causes a downstream shift (Fig. 4*E*). Interestingly and in accord with the downstream shift in poly(A) sites, Fast and Faster Pol II derivatives exhibit a downstream shift in pauses (Fig. 4*F*). This downstream shift in Pol II pausing may reflect inefficient pausing as Pol II travels down the 3′UTR. However, the upstream poly(A) shift in the Slow Pol II strain is not accompanied by upstream-shifted pause locations, but rather by a downstream shift (Fig. 4*F*). Thus, Pol II pausing patterns do not directly correlate with poly(A) profiles for yeast Pol II derivatives with altered elongation rates.

### Spatial relationship between Pol II pauses and poly(A) sites

**Figure 5:**
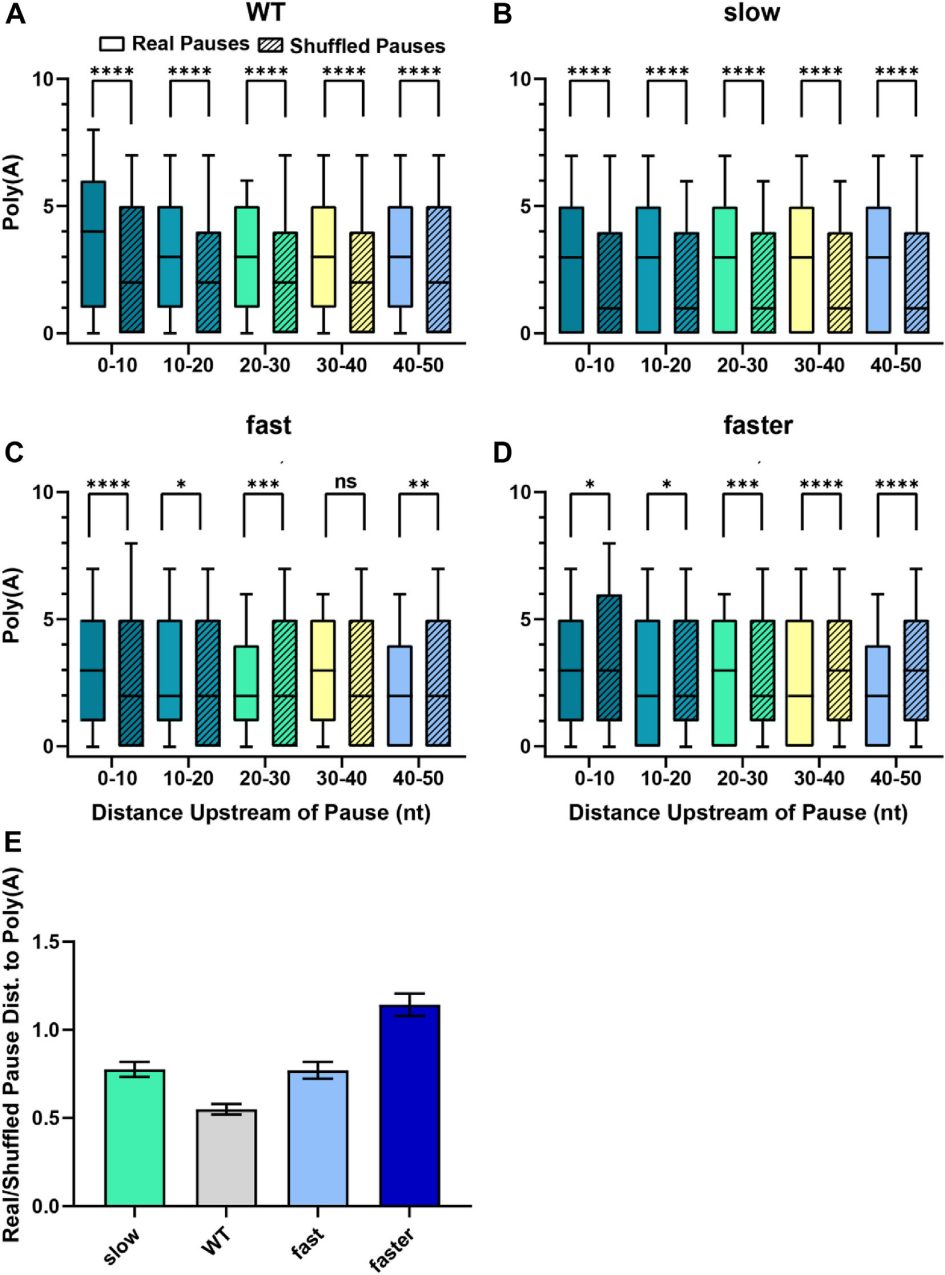
**Polyadenylation is associated with downstream Pol II pausing**. *A–D*, number of poly(A) sites across 10 nt bins 0 to 50 nt upstream to real 3′ UTR-localized pauses (solid color) or shuffled pauses (striped pattern) for yeast strains expressing the indicated Pol II derivative. Boxplots show *upper/lower* quartiles and median with whiskers representing the minimum and maximum values. Statistical significance was assessed by Kolomogorov-Smirnov test: n.s. *p* > 0.05; ∗*p* < 0.05; ∗∗*p* < 0.01; ∗∗∗*p* < 0.001; ∗∗∗∗*p* < 0.0001. *E*, the average distance between a Pol II pause site in the 3′ UTR and the nearest upstream polyadenylation site expressed as a ratio between real and shuffled pause positions. Error bars represent standard error.

Because Pol II must travel past (downstream of) poly(A) sites to permit cleavage and polyadenylation, we addressed a potential spatial relationship between Pol II pause sites and upstream poly(A) sites. To quantitatively define this spatial relationship, we measured the number of poly(A) sites in 10 nt windows from 0 to 50 nt upstream of each Pol II pause site (Fig. 5, *A*–*D*) as well as the minimal distance between each pause and the nearest upstream poly(A) site (Fig. 5*E*). To avoid bias associated with pause-calling, we performed the same analyses using the same number of shuffled pauses that were randomly distributed through the same 3′ UTRs.

In wild-type cells (Fig. 5*A*), poly(A) sites are significantly overrepresented in 10-nt windows from 0 to 50 nt upstream of pauses compared to shuffled control pauses (*p* < 0.001, Kolmogorov-Smirnov test), suggesting that Pol II dwell time affects 3′ end processing. This pattern is upheld in the Slow strain (Fig. 5*B*), but it is less pronounced for the Fast and Faster strains where pausing appears less correlated to poly(A) sites (Fig. 5, *C* and *D*). The distance between Pol II pauses and poly(A) sites is lower in the wild-type strain compared to all three Rpb1 mutant strains (Fig. 5*E*). These observations suggest that although Pol II dwell times are related to polyadenylation, shifts in Pol II pauses are not sufficient to redistribute poly(A) sites.

### Genomic features associated with Pol II pausing in 3′UTRs

We used a random forest classifier (RFC) to assess how well poly(A) sites and other genomic features (DNA sequence and shape, chromatin landscape, Pol II CTD modifications) predict Pol II pause positions. These genomic features have been used to predict pauses in genes (19), but the analysis here is restricted to 3′ UTRs and limited to genes that pass the coverage threshold (>2 reads per nt) in both wild-type and the Rpb1 mutant strains. The 150 genes that pass the coverage threshold are heavily overrepresented for cytoplasmic translation and ribosome biogenesis (4.2 × 10^−110^ and 2.7 × 10^−35^ FDR-adjusted *p*-values, respectively).

**Figure 6:**
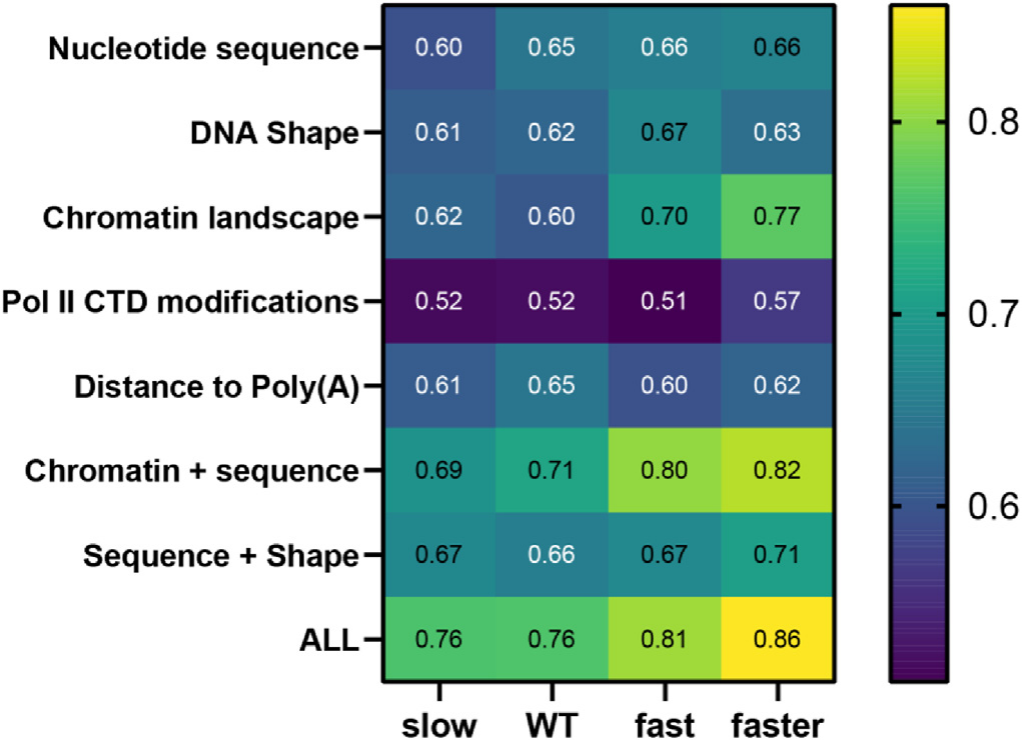
**Genomic features are associated with Pol II pausing in the 3′ UTR.** A random forest classifier was trained to perform a binary classification between real and randomly generated shuffled pauses using underlying sequence information or genomic features at each position. The predictive value of each set of features is expressed as an area under the curve (AUC) of the receiver operating characteristic (ROC), where a value of 0.5 represents a random classification. Analysis was restricted to genes that passed the coverage threshold in mutant strains as well as wild-type (n = 150) and also restricted to positions within 3′ UTRs. The RFC was trained on 75% of the loci and 25% of the loci were used to test model performance. The genomic features that comprise each feature category are listed in Fig. S2.

We trained an RFC to make the binary distinction between *bona fide* pauses and randomly generated shuffled pauses in 3′ UTRs. The area under the curve (AUC) for each receiver operating characteristic (ROC) curve was visualized as a heatmap (Fig. 6). For calling Pol II pauses, poly(A) sites have similar predictive value (AUCs ≈ 0.60) as sequence context or DNA shape. All Rpb1 mutant strains had similar AUCs with respect to distance of the pause to the poly(A) site. However, the number of poly(A) sites within 10 nt of a putative pause was more important for the wild-type strain as opposed to mutant strains (Fig. S3). Consistent with previous reports, Pol II CTD modifications at Ser2, Ser5, and Ser7 were the least predictive feature category (19). This is expected because Pol II is phosphorylated at Ser2 throughout the 3′ UTR where essentially all poly(A) sites are located, whereas Ser5 and Ser7 phosphorylation peaks near the initiation site (22). Interestingly, chromatin landscape AUCs varied directly with elongation speed (Fig. 6), reaching maximal predictive value in the Faster strain (AUC = 0.77).

### Transcription factors impact pausing in the 3′ UTR differently from Rpb1 speed mutants

**Figure 7:**
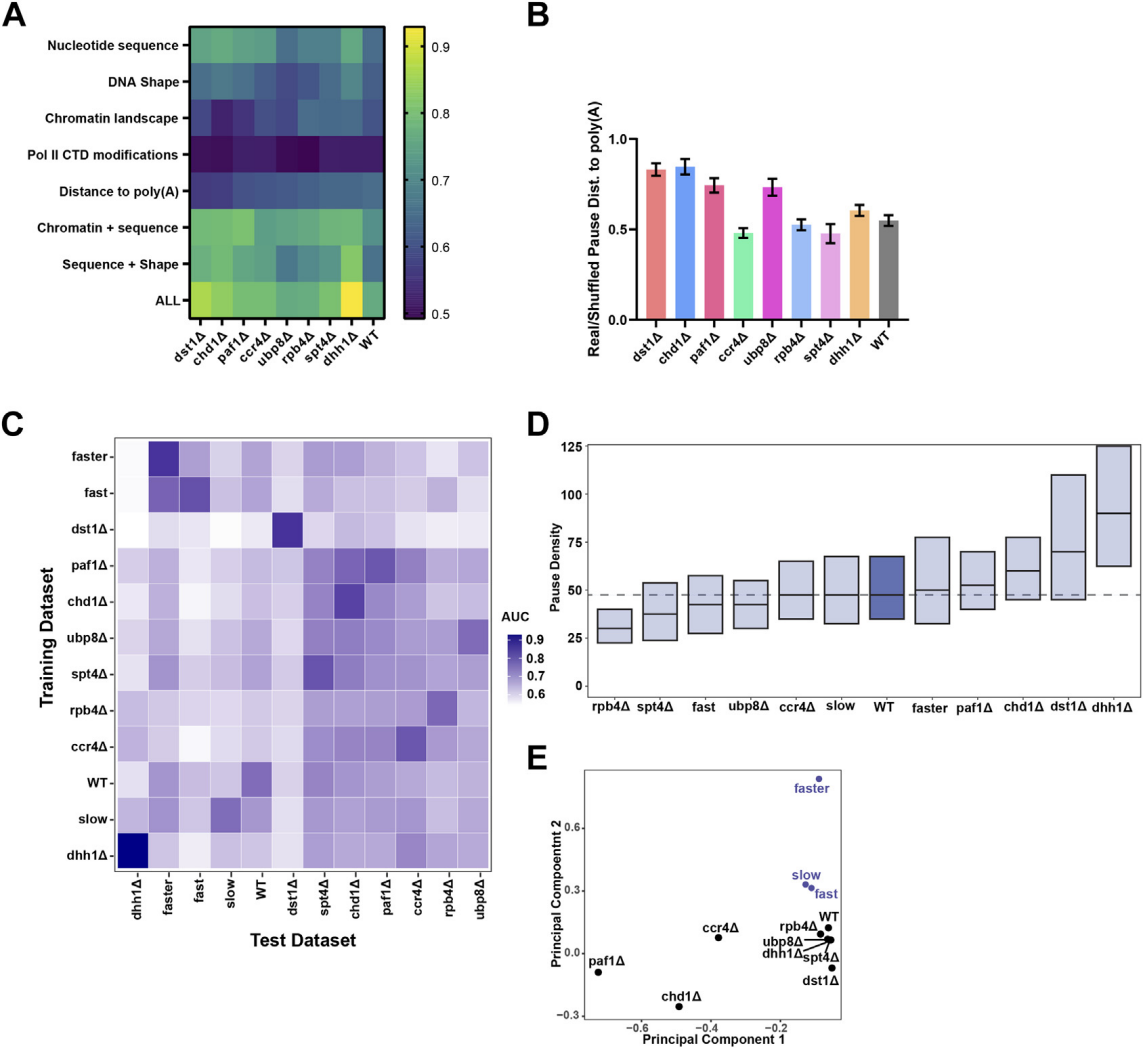
**Transcription factor deletion can disrupt the relationship between Pol II pausing and polyadenylation**. *A*, Heatmap showing AUC for binary classification of real and shuffled 3′ UTR pauses across transcription factor deletion strains. Strains are ordered from lowest (0.56) to highest (0.65) AUC value in the “Distance to poly(A)” feature category. Only high-coverage genes which pass coverage threshold in both mutant and wild-type strains were analyzed. *B*, ratios of the distance between a 3′ UTR pause and its nearest upstream poly(A) site between real and shuffled pauses in transcription factor deletion strains ordered as in (*A*). *C*, Heatmap of AUC values derived by training an RFC on all 3′ UTR pauses from one strain (y-axis) and testing the model on a different strain (x-axis) in a pairwise manner. Comparisons involving only one strain were made by training the RFC on 75% of the pauses and testing on the remaining 25% to evaluate performance. Data are hierarchically clustered across both x- and y-axes. Analysis was limited to genes that passed the coverage threshold in both the wild-type and the mutant strain. *D*, boxplots of pause density (number of pauses per kilobase) in the 3′UTR. The dashed line represents the median pause density of wild-type genes. *E*, principal component analysis based on Pol II pauses in the 3′ UTR of mutant strains. More common 3′ pause sites result in greater proximity between points on this plot.

The impact of transcription factors on Pol II dynamics has been evaluated using an RFC trained on genomic features in wild-type yeast cells (19). Here, we extend these analyses by comparing NET-seq to 3′READs data to understand what factors govern Pol II pausing in the 3′ UTR (Fig. 7*A*). Binary classification of Pol II pausing sites to the distance to the poly(A) sites across seven transcription factor knockout strains show a range of AUC values from 0.56 (*dst1Δ*; least predictive) to 0.65 in (wild-type; most predictive). In strains in which the distance to the poly(A) site is least predictive of Pol II pausing (*dst1Δ*, *chd1Δ*), the average distance between Pol II pauses and the nearest poly(A) sites are most similar to shuffled control (ratio ∼ 0.8); Fig. 7*B*). In general, lower AUC values in the “distance to poly(A)” category correlate with higher real/shuffled ratios of distance to poly(A) site.

We used Pol II pauses from each strain to train an RFC to predict pauses from every other knockout or Pol II mutant strain (Fig. 7*C*). As expected, each strain was a better training set for itself than for any other strain. Strains such as *dst1Δ*, which exhibit a global downstream shift in pausing compared to wild-type (19), are poor training data for predicting pauses in other strains. Conversely, Fast and Faster Pol II mutants, which have very similar transcriptional signatures, provide better training data for each other than for other strains. We note that Fast and Faster Rpb1 mutants show similar pausing patterns according to RFC analysis although they have dissimilar pause densities or total pauses per kb (Fig. 7*D*). These results indicate that the Fast and Faster Pol II have similar pausing patterns due to increased Pol II speed. Pausing in Rpb1 speed mutants is also set apart from wild-type and transcription factor knockouts by principal component analysis (Fig. 7*E*). Some knockout strains (*rpb4Δ*, *ubp8Δ*, *dhh1Δ*, *spt4Δ*) cluster very closely with the wild-type strain along the first two principal components, indicating relatively similar pause sites despite a change in pausing density (Fig. 7*D*).

## Discussion

### Transcriptional elongation rate affects Pol II pausing in 3′UTRs

Co-transcriptional processes are spatiotemporally coupled to the rate of Pol II elongation (4, 23). Yeast Pol II derivatives with slow or fast elongation rates cause upstream or downstream shifts in poly(A) sites, respectively (3). Poly(A) site shifts, and hence the link between Pol II elongation and polyadenylation, occur between 3′ isoforms including at the nucleotide level (5). Pol II occupancy increases downstream of the most abundant poly(A) sites in 3′UTRs (24, 25, 26, 27), indicating that polyadenylation is linked to Pol II slowdown. These observations strongly suggest that Pol II dwell time at the nucleotide-level affects the relative usage of poly(A) sites and that spatial coupling results in the cleavage/polyadenylation complex acting rapidly upon emergence of the nascent RNA from the Pol II elongation complex (5). However, the relationship between Pol II dwell time and poly(A) site selection has not been directly examined.

Here, we investigate the effect of Pol II speed on pausing as well as the relationship between Pol II pausing and poly(A) sites. By definition, Pol II speed mutants increase (Slow Pol II) or decrease (Fast Pol II) pausing on an overall basis, but it is difficult to measure absolute pausing rates. Consequently, our experiments measure relative levels of Pol II pausing at individual nucleotide locations within the 3′UTR, thereby generating a Pol II pausing profile.

Pol II pausing in the Pol II speed mutants can occur at different sequences than in the wild-type strain. Trinucleotide sequence analysis at the Pol II pause site (positions −1, 0, and +1) indicates subtly different nucleotide preferences of the mutant Pol II derivatives as compared to wild-type Pol II. In contrast, nucleotides flanking Pol II pause sites (position −4 to +4) show sequence preferences that are similar among the Pol II derivatives tested. Interestingly, pausing by the Fast and Faster Pol II derivatives shows a downstream shift analogous to the downstream shift in poly(A) sites, but pausing by the Slow Pol II derivative also shows a downstream shift.

These observations indicate that Pol II elongation rate affects the Pol II pausing pattern. This was not a foregone conclusion, as one might have expected that Pol II speed would have comparable effects on dwell time at each nucleotide position and hence no change in relative levels of pausing. Moreover, the change in pausing pattern does not appear to simply reflect a redistribution of pausing sites, as is seen with poly(A) sites. These observations indicate that, unlike poly(A) sites generated by the cleavage/polyadenylation complex, Pol II pause sites are not intrinsically determined by the Pol II elongation machinery.

There are two classes of explanations, not mutually exclusive, for the generation of new pausing sites in the Pol II elongation rate mutants. First, Pol II speed *per se* might have differential pausing effects at individual or clustered nucleotide positions. The similar pattern, both the downstream shift and the new positions, of the Fast and Faster Pol II mutants is consistent with this idea. Second, the Pol II speed mutants might affect sequence specificity (or other aspects of elongation such as effects on chromatin or functional interaction with elongation factors). The mutations examined here are located in the Pol II trigger loop, which affects entry to and stability of the nucleotide precursor at the active site (28, 29, 30). Consistent with this idea, these trigger loop mutations alter sequence preferences at the pause site but not at flanking positions.

### A complex relationship between Pol II pausing and polyadenylation

In all strains tested, Pol II pauses are non-randomly distributed with respect to poly(A) sites. More importantly, the wild-type strain shows a strong co-occurrence of pauses and poly(A) sites 50 to 150 nt downstream of the stop codon as well as a spatial relationship between Pol II pause sites and poly(A) sites. Poly(A) sites are significantly overrepresented 0 to 50 nt upstream of Pol II pauses compared to shuffled pauses, suggesting that Pol II dwell time can impact where polyadenylation occurs. However, Pol II pause and poly(A) sites co-occur less frequently in strains with altered Pol II elongation rates. In addition, slow Pol II elongation leads to upstream-shifted poly(A) sites, whereas Pol II pause sites are shifted further downstream. In Fast and Faster Pol II strains, poly(A) sites are less enriched upstream of pauses, consistent with the decreased overlap between pause and poly(A) sites.

Despite a reduced co-occurrence of pauses with polyadenylation sites, RFC analysis shows a similar predictive value for poly(A) sites as features across wild-type yeast and Pol II derivatives with altered elongation rate (AUC = 0.61–0.65; Fig. 6). However, in the wild-type Pol II strain, the number of poly(A) sites within 10 nt upstream of the pause site is more predictive than in the Slow, Fast, and Faster strains, relative to other features, consistent with a more direct relationship between pausing and polyadenylation in the wild-type strain. Finally, transcription factors related to elongation (Ccr4, Dhh1, Dst1, Paf1, Rpb4, Spt4, and Ubp8) and chromatin remodeling (Chd1) modulate the relationship between alternative polyadenylation and Pol II dynamics. Taken together, these results indicate that Pol II pausing correlates with polyadenylation, but shifts in pausing positions are imperfect predictors of poly(A) site usage.

## Experimental procedures

### Yeast strains

Transcription factor deletion strains ubp8Δ and rpb4Δ used to generate 3′ READs data for this study were the same as in (19). RPO21 mutants were derived from previously reported strains in (3) modified using standard PCR-based methods and lithium acetate transformation to express C-terminal 3x-FLAG epitope tagged Rpb3 from its endogenous locus.

### NET-seq library preparation

Native elongating Pol II RNA-protein complexes were isolated from each strain in biological duplicate essentially as described in (31). Briefly, cells were grown with shaking at 30 °C to mid-log phase (OD_600_ = 0.5–0.8) in YPD medium (2% Bacto Peptone, 1% yeast extract (Difco Laboratories), 2% dextrose), isolated by filtration, and flash-frozen in liquid nitrogen. Frozen cell pellets were cryogenically lysed by six three-minute cycles of grinding in a cryomill (RETSCH). Pol II-RNA complexes were affinity purified using anti-FLAG agarose slurry (Sigma Aldrich, A2220). Isolation of nascent RNA and library construction was performed as described in (19). Library concentration and size distribution was determined using a 2100 Bioanalyzer (Agilent) and qPCR quantitation with library-specific primer (TCCGACGATCATTGATGGTGCCTACAG). 150-bp single-read sequencing was performed on the Illumina Next-seq 500 using the Mid flow cell.

### NET-seq data pre-processing and alignment

NET-seq data from three elongation rate genotypes and one wild-type control was generated in this study (Fig, 1A). Additional, previously published NET-seq data from eight transcription factor knockouts (*ccr4Δ*, *chd1Δ*, *dhh1Δ*, *dst1Δ*, *paf1Δ*, *rpb4Δ*, *spt4Δ*, and *ubp8Δ*) was analyzed (19). Raw sequencing data was pre-processed and aligned as previously described (19). In summary, adapter sequences were removed using cutadapt (32) and fastq files were filtered using PRINSEQ (http://prinseq.sourceforge.net/). Filtered reads were mapped to the SacCer3 genome using TopHat2 (33) and reads that were potentially derived from mispriming during reverse transcription were eliminated. Finally, the 3′ ends of the nascent RNA fragments were annotated using a custom python script *via* the HT-seq package (34). Correlations between biological replicates were evaluated *via* two methods. First, correlations were computed by comparing RPKM values at the gene-level as in (19). Additionally, nucleotide-level correlations were computed using parameters bins -bs 1 with the multiBigwigSummary script from deepTools (35). Nucleotide-level correlations were restricted to genomic regions with high sequencing coverage (≥2 reads per nucleotide).

### Analysis of Pol II pauses

Pol II pauses were identified in high-coverage transcription units (≥2 reads per nucleotide). Both sequence coverage and pause locations were evaluated using read counts summed across two biological replicates. Sequence coverage was evaluated using previously defined transcription units as a reference (36). Transcription units were modified to include genomic coordinates 400 nucleotides downstream of each gene’s stop codon prior to pause-calling. Pauses were defined as nucleotides with sequence coverage of at least 2 reads and at least 3 standard deviations higher than the mean sequence coverage in the surrounding 200 nucleotides. Mean and standard deviation were computed by fitting the read counts at each position to a negative binomial distribution, as described previously (19).

Weighted average 3′ UTR pause positions were computed by taking the product of the pause position (relative to the stop codon) and the number of reads mapping to that pause position, summing these products for all pause positions in a gene’s 3′ UTR and dividing by the read total.

For each pause *in vivo*, the pause trinucleotide is defined by upstream −1 nucleotide, the 0 pause nucleotide, and the +1 downstream nucleotide. The same definition is used for an equivalent number of shuffled pauses, which were restricted to the same transcription units. The number of true *in vivo* pauses divided by the shuffled pauses at each trinucleotide is called the normalized nucleotide preference. Trinucleotide preferences were assessed for each strain and separately between the gene body and 3′UTR in each strain. Normalized trinucleotide preferences at each sequence were compared using a Pearson correlation coefficient. A second set of shuffled pauses was generated as a control and analyzed in the same way as real pauses. Additionally, trinucleotides two to four nucleotides from the pause site were analyzed.

Pause density was calculated as the total number of pauses in the 400 nucleotides downstream of the stop codon. Pause density is expressed as pauses per kilobase. Principal component analysis was used to visualize similarities in pausing across Pol II mutants and transcription factor knockout strains along the first and second principal components.

### Pause analysis with a random forest classifier

Random forest classifier analysis was carried out as described previously (19) with the following modifications. Pause analysis was restricted to the 3′ UTR, defined as the 400 nucleotides downstream of the stop codon for each gene that passed the minimal coverage threshold. Equal numbers of shuffled control loci were generated for each gene, but because pause analysis was restricted to the 3′ UTR, the number of shuffled pauses that were localized to the 3′ UTR were similar but not equal to the number of real pauses. The feature category defined as “Gene” in (19) was replaced by features relating specifically to polyadenylation: distance to the nearest upstream polyadenylation site and the quantity of polyadenylation sites located 0 to 10, 10 to 20, 20 to 30, 30 to 40, and 40 to 50 nucleotides upstream of each pause. Polyadenylation data was collected for each strain individually.

Optimized parameters for training a random classifier on all features were evaluated using the wild-type dataset as described previously (19) and used to analyze the remaining datasets (final parameters: mtry = 5, ntrees = 2000). Only high-coverage genes in mutant strains that also passed the coverage threshold in the wild-type strain were used for analysis.

### 3′ READS library preparation

3′ READS data derived from *rpb4Δ* and *ubp8Δ* strains were generated as part of this study. Cells were grown to mid-log phase (OD_600_ = 0.5–0.8) in YPD with shaking at 30 °C, Cells were lysed and RNA was prepared using the hot acid phenol method (37). RNA was then treated with DNAse I (NEB), and re-purified *via* Qiagen RNeasy kit according to manufacturer’s instructions. Polyadenylated fragments were isolated from 25 μg of total RNA starting material and 18 cycles of final PCR amplification according to the protocol in (38). Library fragment size and concentration was determined using an Agilent 2100 Bioanalyzer. Paired-end sequencing was performed using the Illumina Novaseq platform with the SP flowcell.

### 3′ READS data analysis

Polyadenylation sites specific to each strain were identified using 3′ READS sequencing (14). Data was pre-processed using Python 3 scripts (www.python.org) according to previously published protocols (16). Briefly, T nucleotides at the 5′ end of the R1 read were counted and trimmed from each R1 read. The total number of Ts was then appended to each read ID. Since Ts at the 5′ of the R1 read correspond to potentially polyA-derived nucleotides, reads that did not begin with T were omitted from further analysis. The following 17 bases were aligned to the SacCer3 genome using bowtie with parameters disallowing mismatches and non-uniquely mapping reads (39). True polyadenylation sites were then identified by comparing the number of appended T nucleotides associated with each bowtie-mapped read to the number of genomically-encoded As at that genomic location. Only reads derived from reverse transcription of non-genomically encoded As were retained for further analysis.

### External datasets

External data sets are available *via* the Gene Expression Omnibus (GEO) as follows: NET-seq for *ccr4Δ*, *chd1Δ*, *dhh1Δ*, *dst1Δ*, *paf1Δ*, *rpb4Δ*, *spt4Δ*, *ubp8Δ*, GSE159603 (19); 3′ READS for WT, slow (RPO21-F1086S), fast (RPO21-L1101S), faster (RPO21-E1103G), and *spt4Δ*, GSE151196 (3); data for RFC features was generated as in (19) from data deposited in GEO under accessions GSE92973 (40), GSE141056 (41), GSE61888 (42), GSE98573 (43).

## Data availability

NET-seq data for WT, Slow (*RPO21-F1086S*), Fast (*RPO21-L1101S*), and Faster (*RPO21-E1103G*) and 3′ READS data for *ubp8Δ* and *rpb4Δ* were generated as part of this project and are available at GEO (accession number GSE234406). 3′ READS data for *ubp8Δ* and *rpb4*Δ strains are available.

## Supporting information

This article contains supporting information.

## Conflict of interest

The authors declare that they have no known competing financial interests or personal relationships that could have appeared to influence the work reported in this paper.
