# Supplementary material for: Elongation rate of RNA polymerase II affects pausing patterns across 3′ UTRs
Source: J Biol Chem. 2023 Sep 24;299(11):105289. doi: 10.1016/j.jbc.2023.105289 (PMC10598743; doi:10.1016/j.jbc.2023.105289)
Supplement: Supplementary information [file mmc1.pdf]

Figure S1

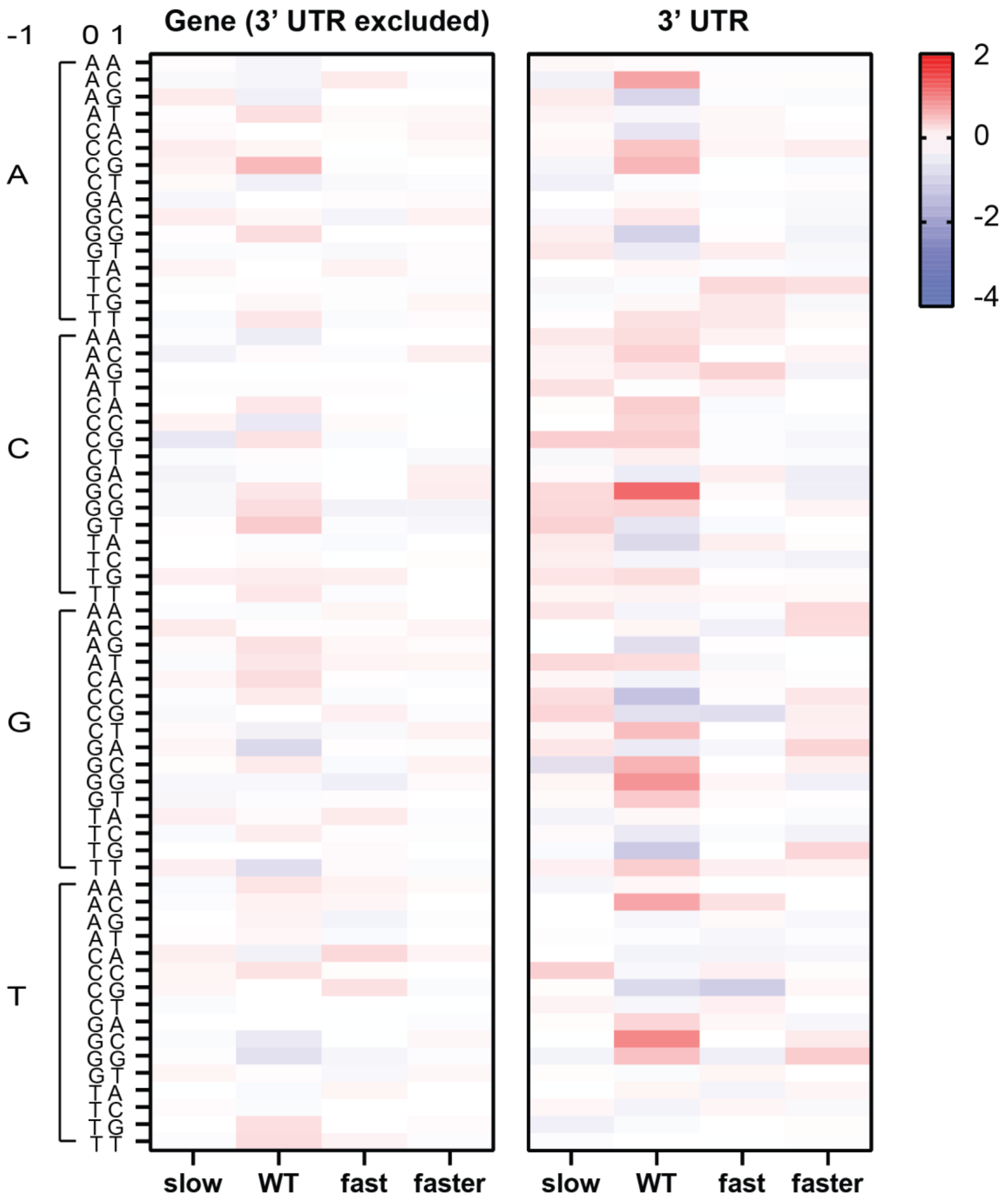

Figure S1. A control showing trinucleotide analysis performed on two sets of shuffled pauses. As a control for the analysis in Fig. 2, real Pol II pauses were shuffled twice. The two sets of shuffled pauses were then compared to each other, rather than to the real pauses from NET-seq analysis. The overall number of pauses remains the same, and is restricted to the same genes and 3' UTRs as the analysis in Fig. 2.

Figure S2

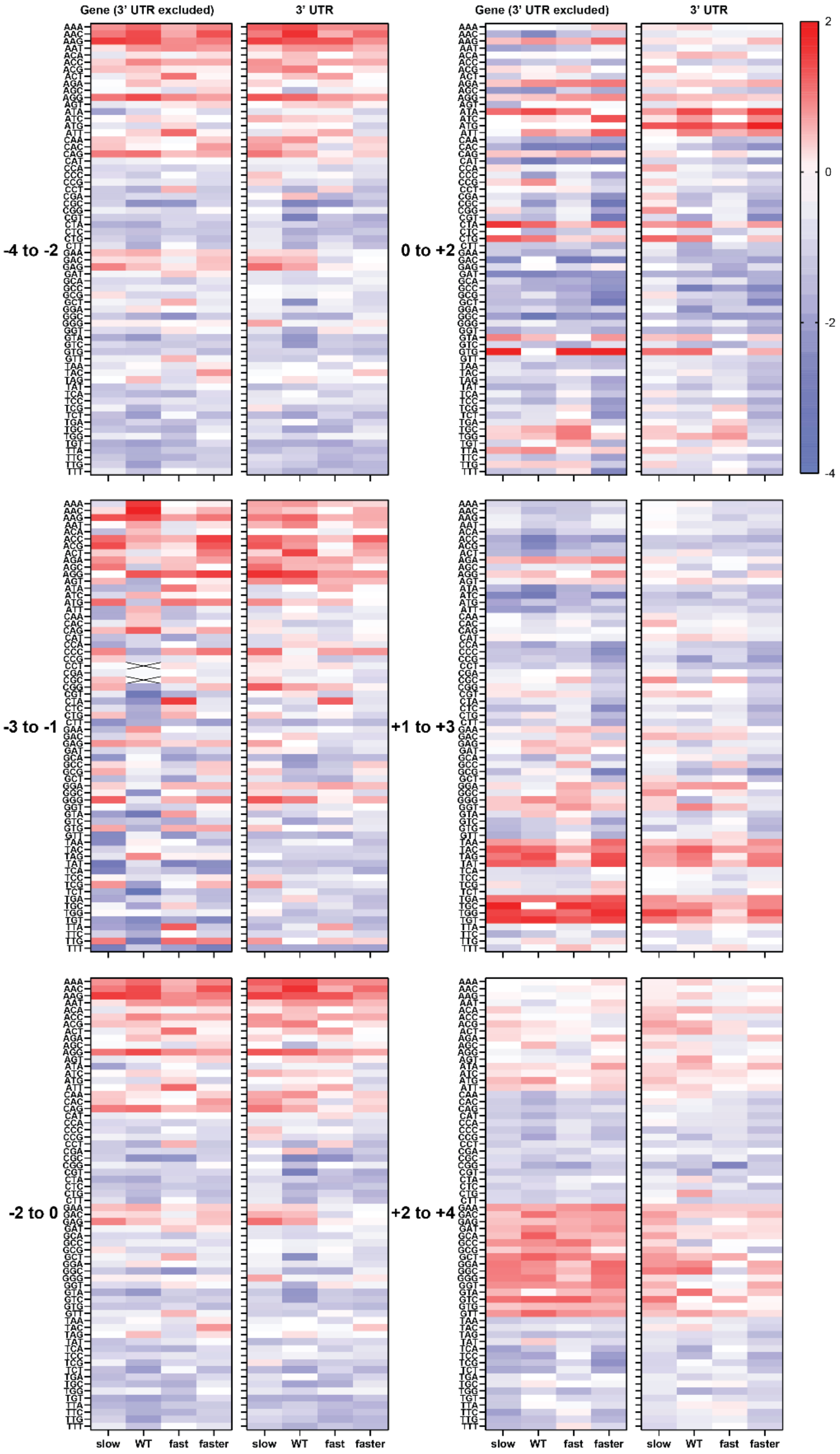

Figure S2. Trinucleotide analysis shown in Fig. 2 extended -4 to +4 up and downstream of the pause site.

Figure S3

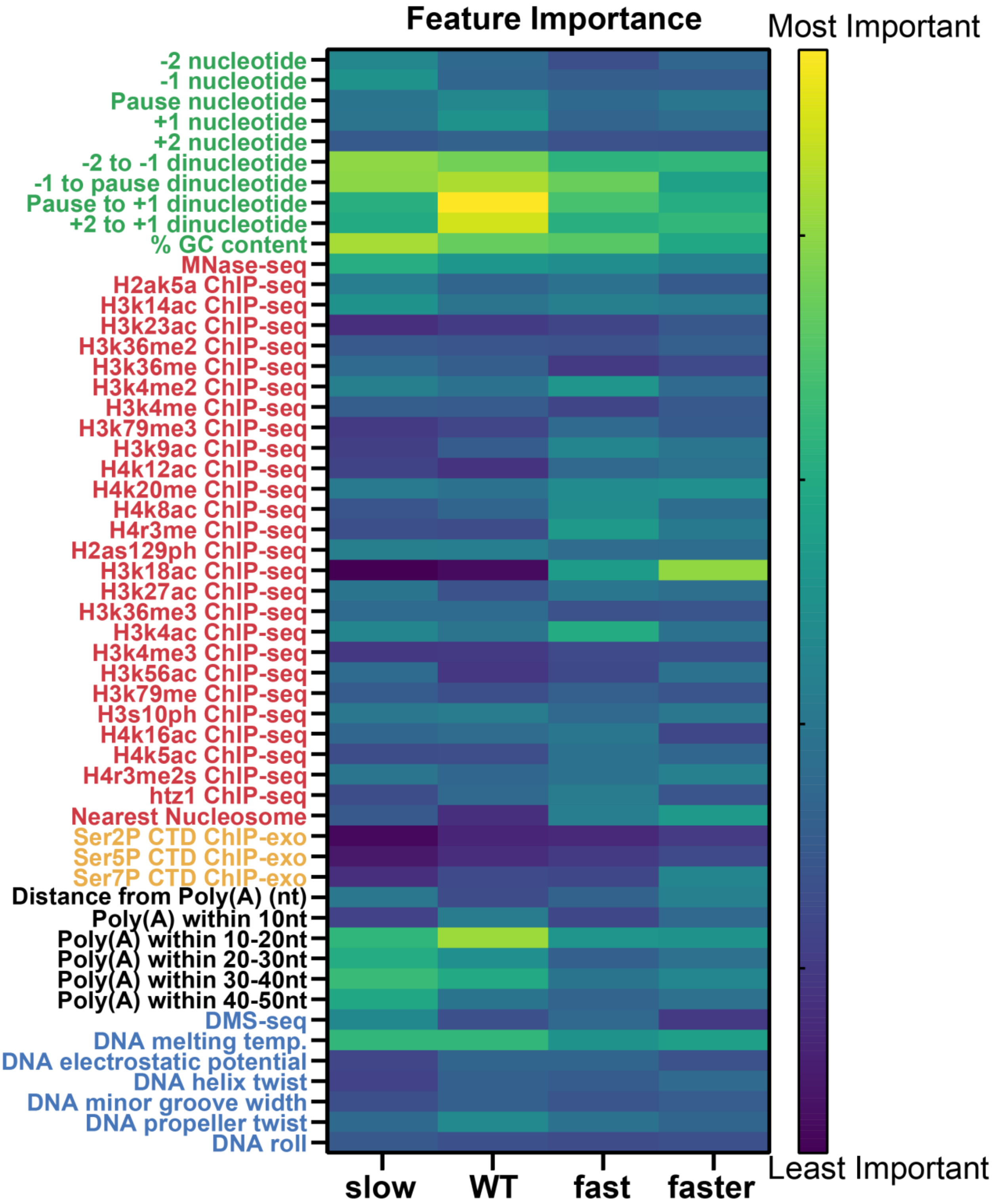

Figure S3. Individual genomic features influence predictive capacity of the RFC to varying extents. Relative importance of each individual feature used to train the RFC, represented as the mean decrease in accuracy upon omission of that feature during

model training. Colors are adjusted such that intensity is kept constant for each strain. Features are color coded by feature category, as green= nucleotide sequence, red= chromatin feature, yellow= Pol II CTD modification, black= distance to poly(A), blue= DNA shape.
